# Effects of residual mulching films with different mulching years on the diversity of soil microbial communities in typical regions

**DOI:** 10.1016/j.heliyon.2022.e12180

**Published:** 2022-12-12

**Authors:** Jianfei Xing, Xufeng Wang, Can Hu, Long Wang, Zhengxin Xu, Xiaowei He, Zaibin Wang, Pengfei Zhao, Qi Liu

**Affiliations:** a College of Mechanical and Electrical Engineering, Tarim University, Alar, 843300, China; b Modern Agricultural Engineering Key Laboratory at Universities of Education Department of Xinjiang Uygur Autonomous Region, Alar, 843300, China; c College of Engineering, China Agricultural University, Beijing, 100083, China; d Institute of Environment and Sustainable Development in Agriculture, Beijing, 100081, China

**Keywords:** Agricultural mulching film, Microplastic, Soil microorganisms, Bacterial diversity, Fungal diversity

## Abstract

Polyethylene mulching film plays a critical role in agricultural production. To clarify the impact of residual film and microplastics on soil microorganisms, this study examined four cotton fields with different film coverage years in typical areas of Xinjiang and analyzed the changes in soil bacterial and fungal community structure and diversity under residual film and microplastics using high-throughput sequencing technology. The results showed that the residual film in the 0–150 mm soil layers and 150–300 mm soil layers at the same sampling point had spatial distribution characteristics of 60–70% and 30–40%, respectively. The short period of the 0–10 years film mulching treatment increased the soil microbial diversity of the cotton field, whereas continuous film mulching for 25 years significantly decreased the soil microbial diversity, in which Proteobacteria was the dominant bacterial phylum and Ascomycetes was the dominant fungal phylum. The microbial diversity of the film-covered soil was lower than that of the control group. The spatial distribution of the residual film and microplastic changed the distribution of the microbial communities. The diversity of the microbial community structure of the 0–150 mm soil layers was higher than that of the 150–300 mm soil layers. The increase in residual film and microplastics had no significant effect on the diversity of the fungal community but decreased the diversity of the soil bacterial community and decreased the relative abundance of Proteobacteria and Campylobacter. In conclusion, long-term film mulching reduced the soil microbial diversity in cotton fields. This study provides a theoretical basis for understanding the impact of film residues on microorganisms and the ecological environment in typical areas.

## Highlights

• The distribution characteristics of soil residual film in cotton field covered by mulch were studied.
• Microbial abundance and diversity were higher in the 0–150 mm soil layer than in the 150–300 mm soil.
• With an increase in plastic films, the overall microbial diversity showed a decreasing trend.
• Plastic film residues are an important factor to reduce soil microbial community diversity in cotton fields.

## Graphical abstract

**Figure:**
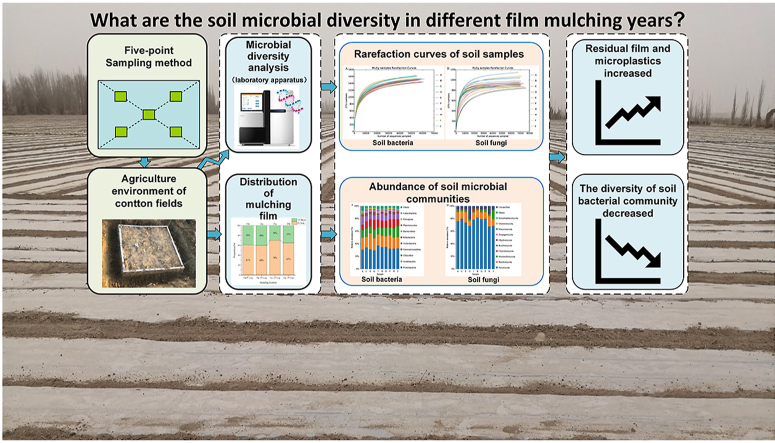

## 1 Introduction

With the increasing use of plastic products, the amount of global plastic waste has increased sharply. Since 1950, the cumulative global plastic production has been approximately 10 billion tons, of which 55% is sent to landfills or discarded environments (Xue et al., 2021). Agricultural plastics include agricultural mulch films, various types of packaging (fertilizers, pesticides, seeds, agricultural products, etc.), and plastic pipes leaked into the soil after use (Nizzetto et al., 2016; Xu et al., 2021a, Xu et al., 2021b, Xu et al., 2021c). As a new agricultural production material applied in the 20th century, mulching films (agricultural plastic films) have remarkably facilitated human agricultural production. Introduced in China more than 50 years ago, plastic film mulching technology has been applied to the planting of crops, such as cotton, corn, and pepper, which has improved agricultural productivity and increased the economic income of farmers (Qi et al., 2021; Liang et al., 2019). Cotton is the main crop planted with mulching films in China. By 2021, the total cotton planting area in China reached 3.169 million hectares. Xinjiang, China's agricultural production and planting base, has a cotton planting area of 2.54 million hectares, accounting for 80.17% of the national cotton planting area (Xinjiang Statistical Yearbook, 2021; China Statistical Yearbook, 2021). With the increased application of mulching films, problems caused by residual mulching films will also arise, although mulching films have brought huge convenience to farmers. Residual mulching films refer to old and shabby mulching films left in the soil that fail to be recycled when seeds are sown. Based on the research results, macroscopically, the residues of mulching films will pollute agricultural ecosystems by destroying the soil structure and affecting the growth of crops. Microscopically, mulching films can further crack and break to form small pieces of films or even microplastics, thereby continuing to act on farmland and influencing animals and microorganisms in the soil.

As a typical area that applies film mulching technology, Xinjiang is facing increasingly obvious problems of residual mulching films caused by the continuous cropping of cotton. Researchers in China and other countries have conducted long-term studies on this topic. According to a study carried out by Yan et al. (2014), residual mulching films in a farmland can decrease soil porosity, hinder the circulation of natural water, and lead to problems such as regeneration salinization in Xinjiang. Parish et al. (2000) found that residual mulching films can prevent seeds from germinating normally, with a decay rate of 8.2%. A study conducted by Niu et al. (2016) showed that the residues of mulching films can increase the mechanical resistance of cultivated land and cause the residual films to entangle, which lowers the quality of soil turning. Hu et al.(2019) investigated the present situation of residual mulching films in Xinjiang from 2016 to 2019, and the results showed that residual mulching films continue to fragment to form microplastics with a diameter of less than 5 mm, posing potential risks to soil fauna and flora. Huang et al. (2020) and Xu et al., 2022a, Xu et al., 2022b, 2023) studied soil microplastics in a Xinjiang cotton field and found that the main component of soil microplastics in the Xinjiang cotton field was PE. The amount of microplastics was related to the time of plastic film covering, which was generally 7851 ± 257 n/kg. Xu et al. (2022a, 2022b) studied whether the plastic film in the soil of the Hebei planting area was the main source of microplastics, and found that the quality of microplastics produced by the plastic film in the cultivated soil was the highest (43.75%). According to a study conducted by Lin et al. (2019), as the amount of residual mulching films increased, the soil moisture content in cotton fields gradually decreased, the average diameter of cotton roots decreased by 82.65%, and the yield of seed cotton declined by 18.5%, which indicates that residual mulching films hinder the growth of cotton roots. Xu et al., 2021a, Xu et al., 2021b, Xu et al., 2021c concluded that the nitrogen content in soil increases as the amount of residual mulching films increases, and the nitrogen content in the soil-root system decreases with an increase in residual mulching films, which ultimately reduces the final yield of cotton. Based on the above studies, the current study mainly focused on the influences of residual mulching films on soil physical and chemical properties, cotton growth, and agricultural machinery.

Soil microorganisms can directly participate in ecological processes such as soil nutrient cycling and the decomposition of organic substances. As an indispensable part of the soil environment, microorganisms affect nutrient absorption by seeds and the growth (Huang et al., 2021; Han et al., 2007; Xu et al., 2021a, Xu et al., 2021b, Xu et al., 2021c). Soil microorganisms are recognized as biological indicators for evaluating the soil environmental quality in terrestrial ecosystems (Liu et al., 2020a, Liu et al., 2020b, 2021), therefore, it is particularly important to study the diversity of soil microorganisms. After exposure to sunlight, ultraviolet radiation, and natural weathering, residual mulch films form plastic fragments or microplastics that penetrate farmland soil, cause changes in the soil environment, and affect the microbial growth (Y. Liu et al., 2020; Zhang et al., 2019a). Wang et al., 2016, Wang et al., 2020a, Wang et al., 2020b conducted a pot experiment, the results of which showed that soil microbial biomass, carbon, nitrogen, enzyme activity, and microbial diversity declined sharply with an increase in the residual quantity of mulching films. Gu et al. (2012, 2014) found that long-term continuous cropping of cotton (10–15 years) can lower the yield of cotton by reducing the diversity of rhizospheric microorganisms (bacteria and fungi). Through a field experiment on planting corn and potatoes with film mulching, Huang (2017) concluded that different amounts of residual mulching films have no significant influence on the diversity of bacteria and fungi in the short term. A study by Blaise et al. (2021) showed that film mulching exerts significant positive effects on soil biological activity, microbial diversity, and abundance. Dong et al. (2017) showed that plastic film mulching can increase the distribution and diversity of soil microorganisms. However, researchers have mainly studied soil microbial diversity under different continuous cropping years and the effect of plastic film on soil microbial rhizosphere diversity through plastic film pot and field simulation experiments. Currently, there are few studies on the diversity of soil microbial structures in cotton fields that have explored mulching film residues. Soil microorganisms are significantly affected by soil environmental factors. Therefore, studying the influences of residual mulching films with different durations on the diversity of soil microbial communities in typical areas is of great significance to further understand the effects of residual mulching films on the farmland environment and ecology, ecological restoration of cotton fields, and pollution control of residual mulching films.

In this study, the soil of cotton fields in typical areas of Xinjiang was selected as the sample, and the high-throughput absolute quantitative method was used to measure the microbial diversity of the soil at different sampling sites. The influence of residual mulching films on microbial diversity was analyzed under different continuous cropping years, providing a theoretical basis for exploring how residual mulching films affect microbial diversity in cotton fields, solving the pollution of residual mulching films, and restoring the ecology. Assuming that residual mulching films have an impact on soil microorganisms in the plow layer, the following aspects were explored: (1) distribution of residual mulching films in different plow layers of the cotton fields, (2) microbial diversity and composition in the soil of cotton fields with and without mulching films, and (3) the effects of mulching films with different mulching years and different tilling depths on biological diversity.

## 2 Materials and methods

### 2.1 Basic information about the sample collection site

Samples were collected from cotton fields (80°30′–81°58′E, 40°22′–40°57′N) in the Alar Reclamation area in the northern part of the Tarim Basin at the southern foot of the Tianshan Mountains, Xinjiang Uygur Autonomous region, Northwest China. Cotton was continuously cropped for 8–25 years. Affected by the extreme continental arid desert climate in the warm temperate zone, the reclamation area enjoys an average annual sunshine of 2556.3–2991.8 h, an average annual temperature of 10.7 °C, a cumulative temperature of 4113 °C, a frost-free period that lasts more than 220 days per year and an average annual precipitation of 40.1–82.5 mm. With abundant sunlight and a strongly stable climate, the reclamation area is a typical cotton-planting area with gray desert soil. According to different cotton planting years, the soil beneath the mulching film in the middle plow layer (0–300 mm) of the same land in this reclamation area was collected as the sample. The soil had the following physical and chemical properties: pH was 8.15 ± 0.17, soil moisture content was 23 ± 3%, soil organic mass was 13.56 ± 3.4 g/kg, alkaline hydrolyzable nitrogen was 56.9 ± 5.3 mg/kg, available phosphorus was 16.89 ± 2.9 mg/kg, and available potassium was 182.7 ± 8.7 mg/kg.

### 2.2 Collection of residual mulching films and soil sampling

After the autumn cotton harvest in October 2021, soil samples and residual films were collected from cotton fields in the agricultural reclamation area of Alar city, Xinjiang. The sampling points were as follows: farm numbers 10, 9, 14, and 12 in Alar City, Xinjiang, China. The plastic film planting years were 10, 16, 20, and 25. A five-point sampling method was used to collect the soil and mulching film samples. All sampling sites were numbered to facilitate the analysis. The soil and mulching films were sampled by layers with sampling depths of 0–150 mm and 150–300 mm, respectively. Table 1 presents the basic information of the sampling sites. For the convenience of understanding, the No. 10 farm samples in the table were represented by A10 numbers, and soil depths of 0–150 mm and 150–300 mm were represented by A101 (A) and A102 (B), respectively. The No. 9 farm samples were represented by B9 numbers, 0–150 mm and 150–300 mm depth soil were represented by B91 (C) and B92 (D), respectively. The No. 14 farm samples were represented by C14 number, 0–150 mm and 150–300 mm depth soil are represented by C141 (E) and C142 (F), respectively, and the No. 12 farm samples were represented by D121 (G) and D122 (H). Control samples were represented by CK number, 0–150 mm, and 150–300 mm depth soil were represented by CK1 (I), and CK2 (J).

**Table 1:** Basic information of soil sampling.

Sampling site	Planting years (a)	Depth	Sample No.	Temperature (°C)	Soil humidity (%)	Altitude (m)	Longitude and latitude
The No. 10 farm	10	0–150 mm	A101 (A)	13°	8.6	1000	81.339914 E
							40.592993 N
		150–300 mm	A102(B)		8.9		
The No. 9 farm	16	0–150 mm	B91(C)	14°	7.2	1018	81.129919 E
							40.587528 N
		150–300 mm	B92(D)		7.8		
The No. 14 farm	20	0–150 mm	C141(E)	15°	7.5	1009	81.779678 E
							40.683219 N
		150–300 mm	C142(F)		7.8		
The No. 12 farm	25	0–150 mm	D121(G)	14°	16.3	1015	81.328216 E
							40.507432 N
		150–300 mm	D152(H)		16.8		
Control	0	0–150 mm	CK1(I)	13°	9.4	1000	81.341445 E
							40.595142 N
		150–300 mm	CK2(J)				

Basic information of the sampling site, including the name of the sampling site, year, number, temperature and humidity, altitude, latitude and longitude.

#### 2.2.1 Collection method of residual mulching films

**Figure 1:**
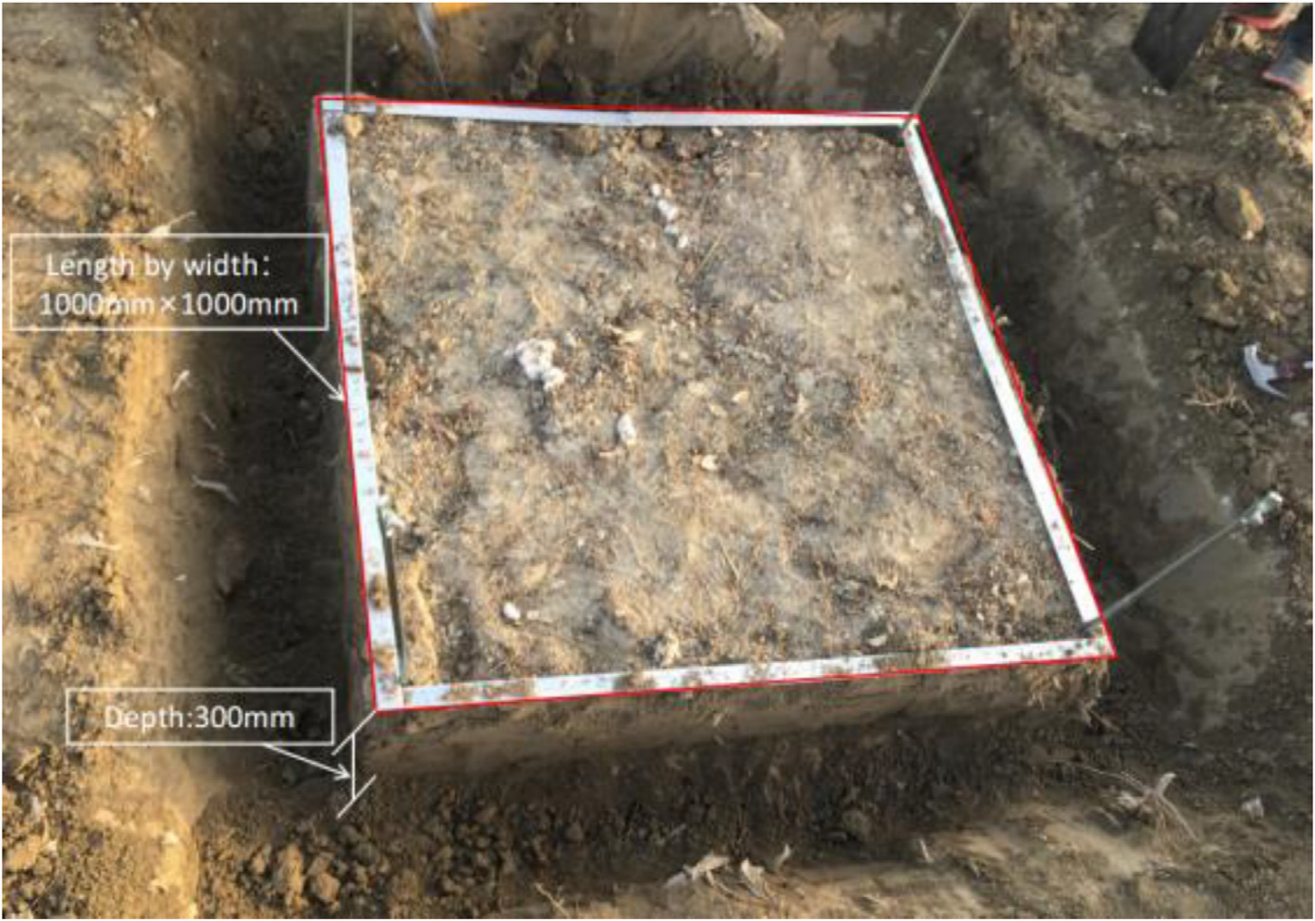
Sampling quadrat of residual plastic film. The length, width, and depth of the sample cubes are 1000 × 1000 × 300 mm respectively.

The residual mulching films were collected from 4 evenly distributed sampling sites where the cotton had been planted for 10–30 years, and all the plots covered an area of more than 3.33 haectares, so the samples were representative. According to the five-point sampling method, the residual mulching films in the 1000 mm × 1000 mm × 300 mm quadrat were manually sampled first in the plow layer with a depth of 0–150 mm and then in the layer with a depth of 150–300 mm (excluding 150 mm). The residual mulching films with an area larger than 0.5 cm^2^ were collected and placed into labeled bags, which were then brought back to the laboratory for processing. Five groups of samples were collected from each cotton field and each group was packaged into 2 bags; thus, there were 40 groups of samples in a single sampling. Figure 1 shows the sampling quadrat of the residual film.

#### 2.2.2 Sampling method of soil

To determine the microbial diversity, it is necessary to collect soil samples from cotton fields. According to the Soil Microbiology Research Specification-I: Methods of collecting soil samples, consistent with the sampling of the residual mulching films, stratified sampling was carried out in the quadrat to collect soil samples, and the cross-sectional layer was observed, as shown in Figure S1. The soil beneath the film was mulched at a depth of 0–150 mm and soil beneath the film mulched at a depth of 150–300 mm was mixed evenly, and the roots and stones were removed. The soil samples were placed into sterile centrifuge tubes and numbered. Each sample was placed in a sterilized sealed bag and stored in an incubator at 4 °C. The samples were sent to the laboratory for DNA extraction. Two groups of soil samples were collected from different plow layers of each cotton field; thus, there were 10 groups of soil samples, including 1 group of soil samples collected from the field without the application of mulching films. Three repeated tests were performed on the 10 groups of soil samples.

### 2.3 Measurement of the amount of residual mulching films and soil microorganisms

#### 2.3.1 Measurement of the amount of residual mulching films at sampling sites

The collected residual mulching films were brought into the laboratory and cleaned twice to remove impurities, such as soil. The residual films were cleaned using a detergent that did not chemically react with the films. The cleaned films were then dried naturally (temperature 25 °C, humidity 42 %). Finally, the cleaned residual films were measured according to the limit and test method for the residual quantity of agricultural mulching films (GB/T 25413-2010). Five groups of residual mulching films collected at depths of 0–150 mm and 150–300 mm at each sampling site were weighed using an electronic scale, and the average weight was taken to obtain the residual quantity of mulching films in each plow layer of the plot. Table S1 shows the distribution of the average amount of residual mulching films at each sampling site.

#### 2.3.2 Total DNA and high-throughput measurement of soil

Microbial diversity research was mainly carried out in the conserved regions of nucleic acid sequences that encode ribosomal RNA. Bacteria were mainly based on the 16S region, and fungi were located in the internal transcribed spacer (ITS) region. After grinding the soil with liquid nitrogen, the MOBIO Power Soil DNA extraction kit was used to extract the total DNA (bacteria and fungi) from the soil samples. The quality and quantity of DNA were evaluated by the ratio of 260 nm/280 nm and 260 nm/230 nm. The DNA was stored at −80 °C for later use. The primer pair (forward primer: 5′-ACTCCTACGGGGAGGGCAGCA-3′; reverse primer: 5′-GGACTACHVGGGTWTCTAAT-3′) amplified the V3–V4 region of the bacterial 16S RNA gene (Bandopadhyay et al., 2020). Primers ITS3F (5′- GCATCGATGAAGAACGCAGC-3′) and ITS4R (5′- TCCTCCGCTTATTGATATGC-3′) were used for PCR amplification of fungal ITS sequences (Yang et al., 2015).

Beijing Biomarker Technologies was used to construct a fragment library on the third-generation Illumina HiSeq 2500 platform (2 × 250 pairs of ends) to sequence and analyze the microbial diversity of the purified DNA samples (www.biocloud.net). The following aspects were analyzed: sequence optimization and quality control, OTU division, taxonomic analysis, single-sample (alpha) diversity analysis, multisample (beta) diversity analysis, intergroup difference analysis, and intersample correlation analysis. All the sequences were submitted to the NCBI SRA database under accession no. PRJNA 869864.

#### 2.3.3 Data analysis and statistics

After the total DNA was extracted from the samples, the primers were designed according to the conserved region. A sequencing connector was added at the end of the primers, and PCR amplification was performed to purify, quantify, and homogenize the products, thereby forming sequencing libraries. Quality inspection was performed on these libraries before sequencing the qualified libraries using an Illumina NovaSeq 6000. First, Trimmomatic v0.33 was used to filter the raw reads obtained by sequencing; second, cutadapt 1.9.1 was applied to identify and remove primer sequences to obtain clean reads without primer sequences. Usearch v10 was used to splice the clean reads of each sample by overlapping and filtering the spliced data according to the length ranges of the different areas. UCHIME v4.2 was used to identify and remove the chimera sequence, thereby obtaining effective reads. Usearch software was used to cluster the reads at a similarity level of 97.0% to obtain the OTUs. Using SILVA as the reference database, the naive Bayes classifier was used to annotate the characteristic sequences and obtain the community composition of each sample counted at various levels (phylum, class, order, family, genus, and species). QIIME was used to generate a table showing species abundance at different classification levels, and a community structure diagram of the samples at different classification levels was drawn using the R language tool. QIIME2 was applied to evaluate the alpha diversity indices of the samples, and microbial diversity was measured using indicators such as Chao1, Ace, Shannon, Simpson, Coverage, and PD_whole_tree. QIIME was used to analyze the beta diversity and compare the similarities of different samples in terms of species diversity (The follow-up analysis in this study was explained by the relative proportion. In the calculation formula, the plastic film-covering group subtracts the control group and then divides it by the control group. The result takes the absolute value and is explained by the relative increase or decrease).

## 3 Results and analysis

### 3.1 Distribution of residual mulching films in different plow layers of cotton fields with different planting years

**Figure 2:**
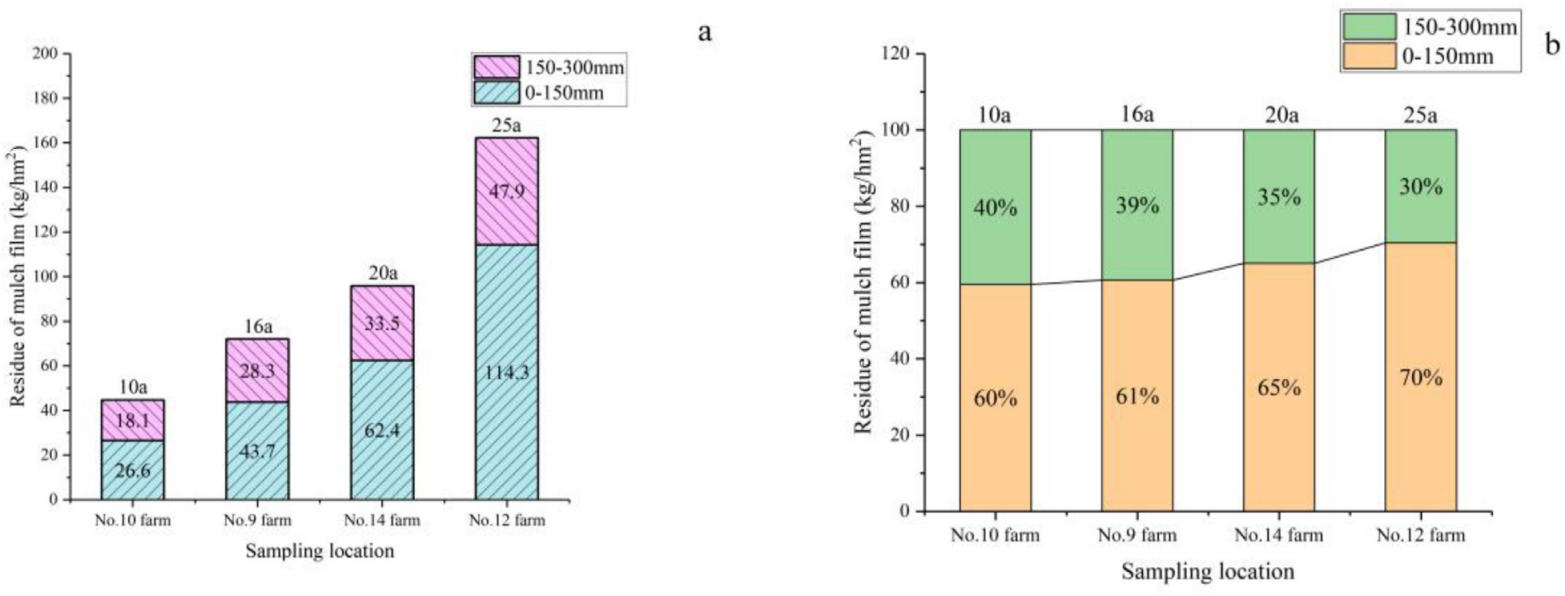
Distribution diagram of residual quantity of mulching films in different plough layers. 10a, 16a, 20a, and 25a indicate the years of mulching on each farm are 10, 16, 20, and 25 years, respectively. (a) Distribution diagram of residual quantity of mulching films in different plough layers. (b) Histogram of proportions of residual mulching films in different plough layers.

Origin was used to process the data in Table S1, and a distribution diagram of the residual mulching films in different plow layers was obtained, as shown in Figure 2.

As shown in Table S1 and Figure 2a, the total residual quantity of mulching films in the 0–300 mm plow layer of cotton fields in the No. 12 farm was 162.21 kg/hm^2^, followed by that in the No. 14 farm (95.9 kg/hm^2^), the No. 9 farm (71 kg/hm^2^) and the No. 10 farm (47 kg/hm^2^). As shown in Figure 2b, the residual quantity of mulching films in the 0–150 mm plow layer was greater than that in the 150–300 mm plow layer of the No. 9, No. 10, No. 14, and No. 12 farms, with the residual mulching films in the 0–150 mm plow layer accounting for 60–70% and those in the 150–300 mm plow layer accounting for 30–40%. The film mulching period increased from 10 to 25 years, and the amount of residual film residues in the farmland gradually increased. Cotton was planted in different continuous cropping years at the four sampling sites. The distribution pattern of residual mulching films and the residual quantity of large films were not affected by the continuous cropping years and mulching years. It is therefore clear that under different continuous cropping years and at different sites, the residual quantity of mulching films in the 0–150 mm plow layer exceeds that in the 150–300 mm plow layers.

### 3.2 Influences of different tilling depths on microbial diversity and community structure of soil

#### 3.2.1 Analysis of microbial diversity of soil with different tilling depths

**Figure 3:**
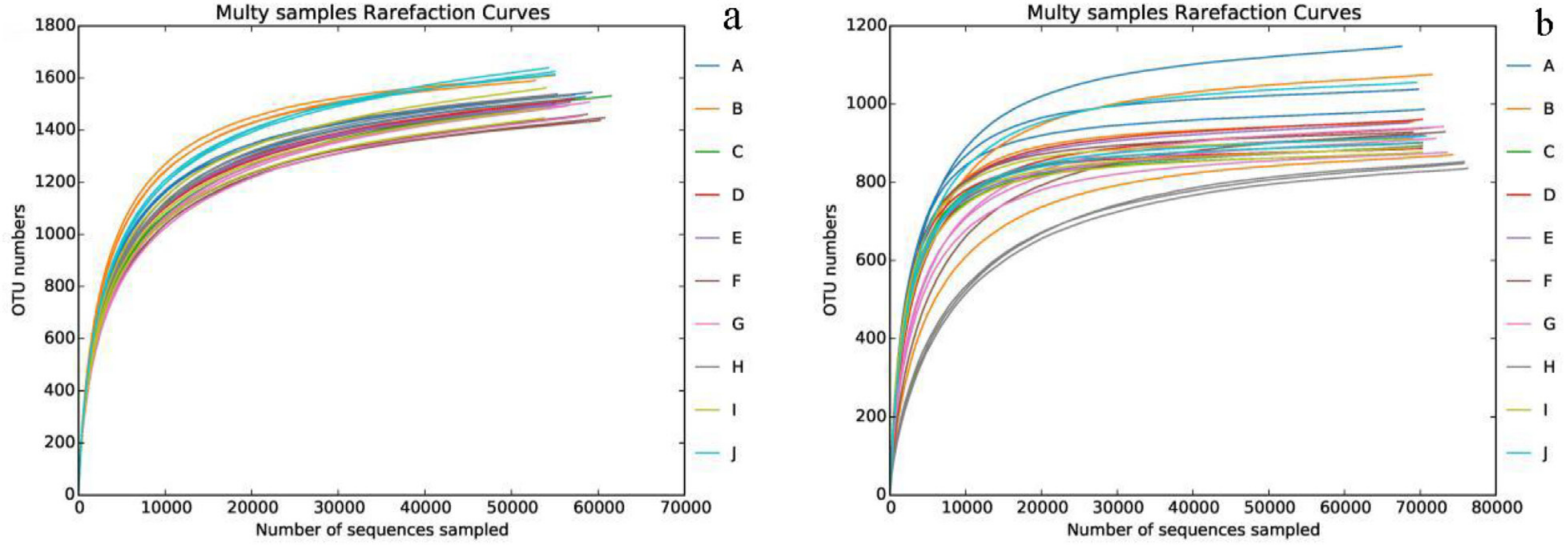
Rarefaction curves of soil samples. (a) Rarefaction curves of soil bacteria; (b) Rarefaction curves of soil fungal.

As shown in Figure 3a and b, the number of OTUs in the soil samples processed in different ways increased with an increase in the number of sequenced samples. When the number of sequenced samples reached 20,000, the curve tended to be flat, and the number of OTUs gradually became saturated, indicating that the measured samples had sufficient sequences covering information about the vast majority of microbial species. Therefore, the samples can accurately reflect the diversity of microbial species in the cotton fields in the Alar Reclamation area. Through an amplicon analysis of 30 soil samples, the obtained sequences were divided into OTUs with a similarity of 97%, obtaining 1450–1629 bacterial OTUs and 846–1058 fungal OTUs. The ACE and Chao indices indicating the abundance of bacterial and fungal communities were 1532–1712 and 1576–1740 and 867–1073 and 868–1117, respectively. The Simpson and Shannon indices of the bacterial and fungal communities were 0.0032–0.0050, 6.09–6.45, 0.0155–0.0994, and 3.65–5.38, respectively.

As shown in Table 2, there were differences in soil microbial diversity and richness between the control group and mulching at 25 years with different tillage depths. In the soil bacterial community, the ACE and Shannon indices of the soil bacterial communities in the 0–150 mm plow layer of the No. 12 farm. were significantly higher than those in the 150–300 mm plow layer (P < 0.05). The richness and diversity of the soil bacterial community in the unfilmed plow layer increased with increasing soil depth. The ACE, Chao, and Shannon indices of the soil bacterial communities in the 150–300 mm plow layer in the control group were significantly higher than those in the 0–150 mm plow layer (P < 0.05), indicating that the abundance and diversity of bacterial communities in the soil at this sampling site improved with increasing soil depth. No significant differences were found in the diversity indices of the other groups (P > 0.05). In the soil fungal community, the Chao index of the fungal communities in the 0–150 mm plow layer of the No. 9 farm was significantly higher than that in the 150–300 mm plow layer (P < 0.05), and the Shannon index of the fungal communities in the 0–150 mm plow layer of the No. 14 farm was considerably higher than that in the 150–300 mm plow layer (P < 0.05). The results showed that the richness and diversity of the soil fungal community decreased with increasing soil depth. There was no significant difference observed among the other groups. In general, the number of OTUs, ACE index, Chao index, and Shannon index of the bacterial communities were higher than those of the fungal communities, implying that the abundance and diversity of the bacterial communities were higher than those of the fungal communities in the cotton planting areas of Alar Reclamation Area, with soil bacterial communities having an obvious advantage. The richness and diversity of the bacterial and fungal communities in the film-covered soil decreased with increasing soil depth.

**Table 2:** Alpha diversity indices of bacteria.

Sample No.	Number of OTUs	ACE index	Chao index	Simpson index	Shannon index
A101	1513.67 ± 17.79bc	1617.51 ± 8.80bc	1646.16 ± 29.77bc	0.0042 ± 0.0003ab	6.20 ± 0.05b
A102	1512.33 ± 6.81bc	1605.22 ± 5.00bc	1633.29 ± 24.25bcd	0.0039 ± 0.0001ab	6.25 ± 0.02b
B91	1537.67 ± 8.02b	1596.88 ± 2.1703bc	1617.30 ± 1.54bcd	0.0047 ± 0.0018a	6.24 ± 0.15b
B92	1604.00 ± 10.58a	1646.43 ± 12.87b	1665.47 ± 17.83b	0.0032 ± 0.0002b	6.45 ± 0.03a
C141	1486.33 ± 28.02cd	1574.03 ± 31.41cd	1596.47 ± 34.43cd	0.0050 ± 0.0001a	6.09 ± 0.02c
C142	1533.67 ± 10.12b	1596.96 ± 10.46bc	1610.31 ± 14.93bcd	0.0040 ± 0001ab	6.24 ± 0.02b
D121	1504.33 ± 4.51bc	1582.60 ± 12.51c	1609.71 ± 21.20bcd	0.0041 ± 0.0001ab	6.23 ± 0.01b
D122	1450.67 ± 12.01d	1532.80 ± 31.49d	1576.28 ± 56.00d	0.0048 ± 0.0001a	6.08 ± 0.02c
CK1	1500.67 ± 59.03bc	1587.46 ± 65.57c	1602.80 ± 61.40bcd	0.0043 ± 0.0006ab	6.20 ± 0.10b
CK2	1629.00 ± 12.53a	1712.00 ± 30.06a	1740.92 ± 37.68a	0.0034 ± 0.0000b	6.37 ± 0.01a

The Alpha diversity index corresponding to each sampling site bacteria respectively, containing OTUs, ACE, Chao, Simpson, Shannon.

#### 3.2.2 Structural analysis of soil microbial communities in different plow layers

**Figure 4:**
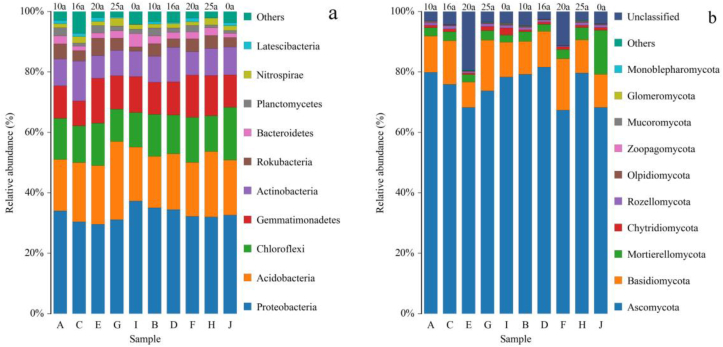
Abundance of soil microbial communities. A, C, E, G, and I represent the communities in the top 10% of phylum level abundance in 0–150 mm soil, and B, D, F, H, and J represent the communities in the top 10% of phylum level abundance in 150–300 mm soil (a) soil bacterial. (b) Soil fungal.

The results regarding the number of OTUs showed that the soil bacterial communities belonged to the 29th phylum, the 92nd class, the 188th order, the 276th family, and the 414th genera and the fungal communities belonged to the 11th phylum, the 36th class, the 83rd order, the 178th family, and the 371st genera. Figure 4 shows the distribution of bacterial and fungal communities with the top 10% relative abundance at phylum level in the 0–300 mm plow layers soil.

Among the soil bacterial communities (Figure 4a), the film-covering age was in the soil of 0–25 a. Proteobacteria, Acidobacteria, Chloroflexi, Gemmatimonadetes, and Actinobacteria were the dominant phyla, with relative abundances reaching 29.58–34.47%, 17.01–21.72%, 10.74–17.52%, 8.32–14.83%, and 7.55–13.17%, respectively. The species composition of the soil samples in the different plow layers was different. Proteobacteria was the dominant phylum among the soil samples processed using the different methods. Among them, the relative abundance of Proteobacteria in the 150–300 mm soil layer of No.9 and No.12 farms, which were covered with plastic film for 16 years and 20 years, was significantly higher than that in the 0–150 mm soil layer (P < 0.05), 13.16% and 9.02% higher, respectively, and the relative abundance of Proteobacteria in the 0–150 mm plow layer of soil samples in the control group was 14.12% higher than that in the 150–300 mm plow layer (P < 0.05). No significant differences were found among the other groups (P > 0.05). Acidobacteria was the second most abundant phylum among all the soil samples processed in different ways. The relative abundance of Agrobacterium in the 0–150 mm soil layer with a film mulching period of 25 years was significantly higher than that in the 150–300 mm soil layer (P < 0.05), and there was no significant difference among the other groups (P > 0.05). Chloroflexi was the third-most abundant phylum. The relative abundance of soil in the 150–300 mm soil layer of the No. 14 farm with a mulching period of 20 years and the control group without mulching was significantly higher than that of the 0–150 mm soil layer (P < 0.05), which was 6.04% and 52.92% higher, respectively; no significant difference was found among the other groups.

Among the fungal communities in the soil (Figure 4b), the plastic film-covered years were in the soil of 0–25 years. Ascomycota, Basidiomycota, and Mortierellomycota were the dominant phyla, with relative abundances reaching 67.36–81.58%, 8.36–17.05%, and 2.31–14.56%, respectively. The species composition of the soil samples varied from one plow layer to another. Ascomycota was the dominant phylum among the soil samples processed in different ways, with its relative abundance in the 0–150 mm plow layer of the control group being 17.74% higher than that in the 150–300 mm plow layer (P < 0.05). There was no significant difference in the relative abundance of Ascomycota between the different plow layers in the other groups (P > 0.05). Basidiomycota was the second most abundant phylum. The relative abundance of Basidiomycota in the 0–150 mm plow layer of soil samples from No.12 farm after 25 years of film mulching was significantly higher than that in the 150–300 mm plow layer (P < 0.05), whereas the relative abundance of Basidiomycota in the 0–150 mm plow layer of soil samples from No. 14 farm after 20 years of film mulching was significantly lower than that in the 150–300 mm plow layers (P < 0.05); there was no significant difference between the other groups. Mortierellomycota was the third-most abundant phylum. No significant difference in the relative abundance of Mortierellomycota was found between the different plow layers of the soil samples processed in different ways (P > 0.05).

#### 3.2.3 Principal coordinate analysis of the structures of microbial communities in different layers of soil

Figure S2 shows the principal coordinate analysis results for the structures of the microbial communities in different layers of soil at the OTU level. As shown in Figure S2-a, the PC1 and PC2 of the bacterial communities were 30.41% and 18.47%, respectively, with a cumulative contribution rate of the two being 48.88%. Except for the soil in the 0–150 mm plow layer in No. 9 farm after 16 years of film mulching, the three repeated microorganisms processed in different ways were strongly clustered with high repeatability. However, the community structures of the microorganisms processed in different ways were significantly different. Figure S2-b shows that the PC1 and PC2 of the fungal communities were 31.85% and 23.97%, respectively, with a cumulative contribution rate of 55.82%. The species composition of the other treatment groups was similar, except that the difference was large in the 150–300 mm soil layer with the film covering the age of 16–25 a. The results showed that the plastic film mulching and the mulching years had a significant impact on the structural characteristics of the bacterial and fungal communities.

### 3.3 Influences of mulching on microorganisms in different layers of soil

#### 3.3.1 Influences of mulching on microbial diversity in different layers of soil

As shown in Tables 2 and 3, film mulching affected the abundance and diversity of the soil microorganisms. Among the bacterial communities, film mulching influenced the number of OTUs and alpha diversity indices of the soil at different depths and sampling points. In the 0–150 mm plow layer, the number of OTUs, ACE index, Chao index, Simpson index, and Shannon index of soil samples collected from the No. 9 farm after 16 years of film mulching were higher than those of soil samples in the control group without mulching film, indicating that film mulching improved the microbial abundance and diversity of soil samples, but the improvement effect was insignificant (P > 0.05). The number of OTUs, ACE index, Chao index and Shannon index of soil samples from No. 10 farm after 16 years of film mulching were higher than those of soil samples from the control group, which implies that film mulching improved the microbial abundance and diversity of soil samples in the No. 10 farm to some extent. The number of OTUs, ACE index, Chao index, and Shannon index of soil samples from No.14 farm after 20 years of film mulching were lower than those of soil samples in the control group without film mulching, which showed that film mulching reduced the microbial abundance and diversity of the surface soil at this sampling point. In the 150–300 mm plow layer, the number of OTUs of soil samples from No. 10, No. 14, and No. 12 farms after 10–25 years of film mulching was significantly smaller than that of soil samples from control group (P < 0.05), with no significant difference found among the other groups. The ACE and Chao indices of the soil samples were ranked from high to low as follows: control group > No. 9 farm Corp > No. 10 farm > No. 14 farm > No. 12 farm, with relevant indices of soil samples in the film covering 10–25 years treatment groups being significantly lower than those of soil samples in the control group without film, indicating that film mulching significantly reduced the microbial abundance of soil in the 150–300 mm plow layer of cotton fields in this area. The Shannon index in the soils of No. 10, No. 14, and No. 12 farms with film mulching years of 10–25 years were significantly lower than those in the control group without film mulching (P < 0.05), which showed that film mulching significantly reduced the soil diversity of cotton fields in this area. In summary, film mulching affected the microbial diversity of soil bacterial communities in cotton fields. There was no significant effect on the microbial abundance and diversity of bacterial communities in the soil of the 0–150 mm plow layer (P > 0.05), but there was a significant decrease in the microbial diversity and abundance of bacterial communities in the 150–300 mm plow layer (P < 0.05).

**Table 3:** Alpha diversity indices of fungal.

Sample No.	Number of OTUs	ACE index	Chao index	Simpson index	Shannon index
A101	889.67 ± 14.01bc	902.62 ± 19.73bc	924.90 ± 29.54bc	0.0206 ± 0.0010a	5.23 ± 0.04a
A102	929.67 ± 38.28bc	942.24 ± 43.44bc	981.88 ± 73.62bc	0.0392 ± 0.0345a	5.09 ± 0.43a
B91	1058.33 ± 82.71a	1073.93 ± 87.76a	1117.41 ± 70.61a	0.0182 ± 0.0026a	5.38 ± 0.06a
B92	967.67 ± 103.58ab	980.09 ± 104.54ab	993.56 ± 112.90b	0.0823 ± 0.1078a	4.61 ± 1.02a
C141	911 ± 33.05bc	924.26 ± 33.40bc	942.67 ± 37.99bc	0.0321 ± 0.0045a	4.73 ± 0.06a
C142	846.00 ± 8.89c	867.71 ± 9.30c	868.92 ± 9.87c	0.0994 ± 0.0108a	3.65 ± 0.09b
D121	922.00 ± 30.51bc	937.48 ± 31.89bc	979.96 ± 50.52bc	0.0155 ± 0.0021a	5.37 ± 0.12a
D122	920.33 ± 15.89bc	931.96 ± 17.91bc	945.64 ± 17.60bc	0.0851 ± 0.1115a	4.71 ± 1.03a
CK1	893.33 ± 18.23bc	903.46 ± 16.68bc	928.13 ± 26.71bc	0.0321 ± 0.0045a	5.16 ± 0.24a
CK2	959.33 ± 84.10b	971.05 ± 83.37bc	986.70 ± 76.23b	0.0994 ± 0.0108a	5.02 ± 0.01a

The Alpha diversity index corresponding to each sampling site fungi respectively, containing OTUs, ACE, Chao, Simpson, Shannon.

Among the fungal communities, in the 0–150 mm plow layer, the number of OTUs in the soil samples ranked from large to small as follows: No. 9 farm > No. 12 farm > No. 14 farm > control group > No. 10 farm. The number of OTUs in the soil samples collected from No. 9 farm after 16 years of film mulching was significantly larger than that of the control group with film, and there was no significant difference among the other treatments (P > 0.05). The ACE and Chao indices of the soil samples varied from high to low as follows: No. 9 farm > No. 12 farm > No. 14 farm > control group > No. 10 farm Corp. The relevant indices of soil samples from No. 9 farm after 16 years of film mulching were being significantly higher than those of soil samples in the control group without film (P < 0.05), which indicating that the film mulching considerably improved the microbial abundance of soil in the 0–150 mm plow layer. In the 150–300 mm plow layer, the number of OTUs in the soil samples was ranked from large to small as follows: No. 9 farm > control group > No. 10 farm > No. 12 farm > No. 14 farm. The number of OTUs in soil samples in the No. 9 farm was significantly larger than that of the control group (P < 0.05), and no significant difference was found among the other treatment groups (P > 0.05). The ACE and Chao indices of soil samples from No. 9 farm after 16 years of film mulching were significantly higher than those in the control group; the ACE and Chao indices of the No. 12 and No. 14 farms with film mulching period of more than 20 years were significantly lower than those of the control group, indicating that the soil microbial richness was significantly increased with continuous film mulching for 16 years but decreased with the increase in film mulching years to 20 years. The Simpson and Shannon indices of the soil samples processed in different ways were not significantly different (P > 0.05), indicating that film mulching did not affect on microbial diversity of soil fungal communities.

#### 3.3.2 Influences of mulching on the structures of microbial communities in different layers of soil

**Figure 5:**
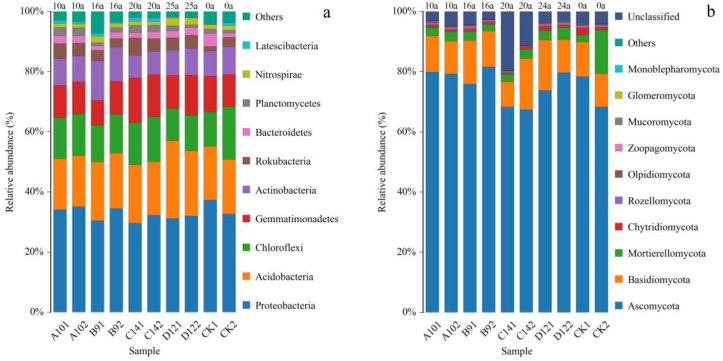
Structure of the soil microbial communities. shows the comparison of the top 10 groups of communities in the bacterial and fungal phylum at each sampling site for 0–150 mm and 150–300 mm soil depths. (a) Soil bacterial. (b) Soil fungal.

Figure 5 shows the influences of film mulching on the structures of microbial communities in soil samples processed using different methods. Distribution of species composition before and after film mulching. As shown in Figure 5a, at the phylum level of the soil bacterial communities, Proteobacteria, Acidobacteria, Chloroflexi, and Gemmatimonadetes were the dominant phyla of bacteria in the control group and the film mulching groups, with relative abundances of 29.58–37.27%, 17.01–25.90%, 10.74–17.52%, and 8.32–14.83%, respectively. In the 0–150 mm plow layer, compared with the control group without film, when film mulching years from 10 to 25 years, the relative abundance of Proteobacteria in soil samples collected from the No. 10, No. 12 and No. 14 farms significantly decreased 18.28%, 16.53%, and 20.63%, respectively (P < 0.05); the relative abundance of Actinobacteria in soil samples in the No. 12 farm Corp after 20 years of film mulching was 25.90%, which was 44.77% significantly higher than that in the control group. The relative abundance of Chloroflexi in soil samples collected from the No. 14 farm after 25 years of film mulching was 14.02%, which was 22.34% higher than that of the control group. Gemmatimonadetes in the soil samples of the No. 9 farm after 16 years of film mulching and the No. 14 farm after 25 years of film mulching had a relative abundance of 14.83% and 8.32%, respectively, which were 29.99% higher and 24.72% lower than that of the control group, respectively, and no significant differences were found among the other treatment groups (P > 0.05). In the 150–300 mm plow layer, with film mulching years 10a to 25a, the relative abundance of Proteobacteria in soil samples from No. 9 farm (34.47%) and the No. 10 farm (35.07%) were 5.53% and 7.38% higher than those of the control group. respectively. The relative abundance of Acidobacteria in the No. 12 farm was 21.72%, which was 19.48% significantly lower than that in the control group. The Chloroflexi in the soil of the No. 10, No. 12, and No. 14 farms had relative abundance of 13.87%, 11.81%, and 14.92%, respectively, which were 20.84%, 32.61%, and 14.84% lower than that of the control group, respectively. The relative abundance of Gemmatimonadetes in the No. 12 farm and No. 14 farms after 20 years and 25 years of film mulching was significantly reduced by 24.32% and 30.62%, respectively (P < 0.05), and there was no significant difference among the other treatment groups (P > 0.05).

As shown in Figure 5b, at the phylum level of the soil fungal communities, Ascomycota, Basidiomycota, and Mortierellomycota were the dominant phyla, with relative abundances of 68.27–81.58%, 8.36–17.05%, and 2.31–14.56%, respectively. In the 0–150 mm plow layer, the relative abundance of Ascomycota in the No. 14 farm after 20 years of film mulching was 68.28%, which was 12.82% significantly lower than that in the control group without film (P < 0.05). Basidiomycota in the soil samples of the No. 9 and No. 12 farms after 16 and 25 years of film mulching had relative abundances of 14.42% and 16.83%, respectively, which were 25.23% and 46.12% significantly higher than that of the control group, respectively. The relative abundance of Basidiomycota in the No. 14 farm after 20 years of film mulching was 27.38% lower than that of the control group, and no significant difference was found among the other treatment groups (P > 0.05). In the 150–300 mm plow layer, the relative abundance of Ascomycota in the soil samples from No. 10, No. 9 and No. 12 farms after 10 years and 16 years and 25 years of film mulching was 79.22%, 81.58%, and 79.65%, respectively, which was 16.05%, 19.50%, and 16.67% significantly higher than that in the control group. Basidiomycota in soil samples from No. 14 farm after 20 years of film mulching had a relative abundance of 17.05%, which was 54.77% significantly higher than that of the control group without film. Film mulching significantly reduced the relative abundance of Mortierellomycota in the soil samples in the No. 9, No. 10, No. 12, and No. 14 farms with film mulching ages of more than 10 years were 83.53%, 84.11%, 78.08%, and 79.27%, respectively.

#### 3.3.3 LefSe analysis of bacterial and fungal communities in soil microorganisms

With LDA = 4 as the threshold value, a linear discriminant analysis was performed on the composition of bacterial and fungal communities in cotton field soil processed using different methods to determine biomarkers related to microbial diversity in cotton field soil. Film mulching resulted in apparently different structures of bacterial and fungal communities in cotton field soil, with significant differences found in 36 bacterial branches and 30 fungal branches. In terms of bacteria (Figure S3-a), there were 7, 9, 12, 6, and 9 significantly different bacterial branches in the soil samples from No. 9, No. 10, No. 12, and No. 14 farms and the control group. After 16 years of film mulching, the soil from No. 9 farm was rich in Rhizobiales and Betaproteobacteria. After 10 years of film mulching, the soil from No. 10 farm was rich in Actinomarinales and Alphaproteobacteria. After 25 years of film mulching, the soil in the No. 12 farm was rich in Pyrinomonadales and Sphingomonas, and after 20 years of film mulching, the soil from No. 14 farm was rich in Gemmatimonadaceae and Rokubacteriales. The soil in the control group was rich in Geminicoccaceae and Tistrellales. In fungi (Figure S3-b), the No. 9, No. 10, No. 12, and No. 14 farms and the control group had 3, 3, 10, 10, and 4 significantly different fungal branches, with the soil in the No. 9 farm after 16 years of film mulching rich in Thelebolales, Pyronemataceae, and Pezizales; the soil in the No. 10 farm after 10 years of film mulching rich in Erysiphe; the soil in the No. 12 farm after 25 years of film mulching rich in Cladosporium, Pleosporaceae, Fusarium, Nectriaceae, Vishniacozyma, Bulleribasidiaceae, and Tremellales; the soil in the No. 14 farm after 20 years of film mulching rich in Purpureocillium, Ophiocordycipitaceae, Hypocreales, and Cephalotrichum, and the soil in the control group rich in Aspergillus.

### 3.4 Influences of different residual quantities of mulching films on soil microorganisms

#### 3.4.1 Influences of different residual quantities of mulching films on soil microbial diversity

Table 4 shows the residual quantities of mulching films, as well as the differences between the alpha diversity indices of bacterial and fungal communities at different sampling sites. With an increase in the film mulching period from 10 to 25 years, the total residual quantity of mulching films in different soil samples was ranked from large to small as follows: D12 > C14 > B9 > A10, with the largest residual quantity of mulching films found in soil samples from No. 12 farm after 25 years of film mulching. In the soil bacterial communities (Table 4a), the number of OTUs, ACE index, and Chao index of soil samples after 25 years of film mulching were significantly lower than those of the control group without film (P < 0.05), which indicated that the residual quantity of mulching films could drastically reduce the microbial abundance of soil bacterial communities, and the microbial abundance of bacterial communities gradually declined as the residual quantity of mulching films increased. Concerning soil fungal communities (Table 4b), although soil sample collected from the No. 12 farm had the largest residual quantity of mulching films, and the number of OTUs, Shannon index, Simpson index, ACE index, and Chao index were not significantly different from those of the control group (P > 0.05). In addition, only the soil sample from No. 9 farm after 16 years of film mulching significantly exceeded that of the control group in terms of the number of OTUs, Simpson index, ACE index, and Chao index (P < 0.05), and there was no significant difference between the diversity dice of the other treatment groups (P > 0.05), indicating that the residual quantity of mulching films did not insignificantly influenced the microbial diversity of the soil fungal communities.

**Table 4:** Influences of different residual quantities of mulching films on soil microbial diversity.

Sample No	Residual quantity of mulching films kg/hm^2^	Number of OTUs	Shannon index	Simpson index	ACE index	Chao index
(a) Bacteria						
A10	44.7	1513 ± 12.07bc	6.22 ± 0.04ab	0.0041 ± 0.0003a	1611.36 ± 9.29abc	1639.72 ± 25.29ab
B9	72	1570.83 ± 37.29a	6.34 ± 0.15a	0.0039 ± 0.0014a	1621.65 ± 28.37ab	1641.39 ± 28.71ab
C14	95.9	1510.00 ± 32.05bc	6.16 ± 0.09b	0.0045 ± 0.0006a	1585.50 ± 24.42bc	1603.39 ± 24.92b
D12	162.2	1477.50 ± 30.50c	6.16 ± 0.08b	0.0045 ± 0.0004a	1557.70 ± 34.69c	1592.99 ± 42.06b
CK	0	1564.83 ± 79.98ab	6.29 ± 0.11ab	0.0039 ± 0.0007a	1649.73 ± 82.06a	1671.86 ± 88.31a
(b) Fungal						
A10	44.7	909.67 ± 33.83b	5.16 ± 0.29a	0.0299 ± 0.0241a	922.43 ± 37.17b	953.39 ± 59.08b
B9	72	1013.00 ± 97.44a	4.99 ± 0.77a	0.0502 ± 0.0768a	1027.01 ± 100.47a	1055.48 ± 108.15a
C14	95.9	878.50 ± 41.67b	4.19 ± 0.60b	0.0658 ± 0.0376a	895.98 ± 37.95b	905.79 ± 47.41b
D12	162.2	921.17 ± 21.78b	5.04 ± 0.75a	0.0503 ± 0.0802a	934.72 ± 23.33b	962.80 ± 38.70b
CK	0	926.33 ± 65.34b	5.09 ± 0.17a	0.0309 ± 0.0095a	937.25 ± 65.28b	957.41 ± 60.32b

A10, B9, C14, D12, and CK represent the sampling points of No. 10 Farm, No. 9 Farm, No.14 Farm, No. 12 Farm, and the control group, respectively. The residual film residue is the average, and the remaining data are average ± SE. values with different lowercase letters in the same column are significantly different at 0.05 level.

The residual film residue is the average, and the remaining data are average ± SE. Values with different lowercase letters in the same column are significantly different at 0.05 level.

#### 3.4.2 Influences of different residual quantities of mulching films on the structures of soil microbial communities

As shown in Figure S4, the community composition of soil microorganisms varied with the change in the residual quantity of mulching films. At the phylum level of bacteria (Figure S4-a), Proteobacteria, Acidobacteria, and Chloroflexi were the dominant phyla in all groups. The relative abundance of the different dominant species varied with changes in the residual quantity of mulching films. The relative abundance of Acidobacteria in the soil sample from No. 12 farm after 16 years of film mulching was 23.73%, which was 39.48%, 24.59%, 27.16%, and 31.57% higher than that of the No. 10, No. 9, and No. 14 farms with film mulching of 10–25 years and the control group, respectively. Proteobacteria and Chloroflexi in the soil sample of the control group had relative abundances of 34.95% and 14.51%, respectively, which were 7.93% and 1.10%, 10.68% and 11.59%, 16.63% and 5.72%, and 28.51% and 0.35% higher than those in the No. 9, No. 10, No. 12, and No. 14 farms after 16a–25a of film mulching, respectively.

At the phylum level of fungi (Figure S4-b), Ascomycota, Basidiomycota and Mortierellomycota were dominant. The relative abundance of Ascomycota in the soil sample of the No. 14 farm was lower than that of the control group without film, whereas the relative abundance of Ascomycota in the soil samples from No. 9, No. 10, and No. 12 farms after 16a, 10 years and 25 years of film mulching was 7.52%, 8.54%, and 4.68% significantly higher than that of the control group, and the relative abundance of Basidiomycota in the soil samples of the No. 9, No. 10, and No. 12 farms was 16.64%, 1.61%, and 23.33% higher, respectively, than those of the control group. However, Mortierellomycota in the soil sample of the control group had relative abundances of 219.17%, 64.64%, 57.75%, and 205.81%, respectively, which were significantly higher than those in the farms with 10–25 years of film mulching. The above results showed that residual mulching films affected the relative abundance of soil fungal communities in cotton fields. The residual quantity of mulching films increased the relative abundance of Ascomycota and Basidiomycota but reduced that of Mortierellomycota.

## 4 Discussion

The pollution caused by residual mulching films in cotton fields in Xinjiang has always been the focus of attention in the agricultural field. Xinjiang is a typical and representative case study in China, which suffers the most serious pollution caused by residual mulching films. Many studies have shown that agricultural mulching film, as an important agricultural production material in typical areas of Xinjiang, preserves heat, soil moisture and optimizes the crop growth environment. However, when the residual quantity of mulching films in farmland exceeded 75 kg/hm^2^, crop growth was adversely affected. Current studies have mainly focused on the influences of different residual quantities of mulching films on soil structure, water and fertilizer migration, and crop growth, but the influences of residual mulching films on the biological characteristics of soil microorganisms has rarely been studied. In this study, soil samples were collected from 4 cotton fields in southern Xinjiang with long-term film mulching. The Illumina HiSeq 2500 platform was used to sequence and analyze the microbial diversity of the purified DNA samples in 30 groups to explore the diversity of soil microbial communities (bacteria and fungi) in different cotton fields and to study the influences of different tilling depths and residual quantities of mulching films on the structures of microbial communities. It was found that mulching films, which have long been used in human labor, can change soil microbial diversity as their residual quantity varies. Additionally, with an increase in mulching duration, the abundance and community structure of soil microorganisms in the different tiller layer residues changed.

The soil samples used in this study were collected from cotton fields in the Alar reclamation area of southern Xinjiang. With a pH value of approximately 8.1–8.7, the soil was saline-alkali, which is consistent with the results of Wu et al. (2013) and Liu et al. (2019). The soil collected at the sampling points was gray desert soil at an altitude of approximately 1000 m. The soil samples were mulched with plastic films for different years; therefore, the residual quantities of mulching films in these soil samples also differed. Therefore, the abundance and community structure of soil bacteria and fungi in the film-covered cotton fields can reflect in the microbial diversity in typical areas.

The analysis results showed that the residual quantity of mulching films in soil samples mulched with films and soil samples in the control group increased with the years of film mulching, showing spatial distribution characteristics. In this study, the residual mulching films in the 0–150 mm plow layer accounted for 60–70%, and those in the 150–300 mm plow layer account for 40–60%. This is consistent with the results of Wang et al. (2018) regarding the distribution characteristics of residual films in cotton fields under drip irrigation conditions in the typical oases in Xinjiang. The residual film was mainly concentrated in the 0–300 mm plow layer of the cotton fields, and the results showed that the residual film mass of the 0–150 mm tillage layer accounted for 62%–67%, and the residual film mass of the 150–300 mm was less, accounting for 33%–38%. This is because of drip irrigation, plowing, soil preparation, and rotary tillage. The mulching films that failed to be removed from the soil surface were plowed to the middle layer during autumn or spring plowing. With the drip irrigation effect, the broken film moved downward; therefore, the 0–150 mm plow layer had a large residual quantity of mulching films. Generally, farmland is plowed to achieve sowing conditions at a depth of 80–150 mm, and there are relatively few residual mulching films in the 150–300 mm plow layer. Therefore, the pollution of residual mulching films in cotton fields is a problem caused by the long-term accumulation of residual mulching films, and such pollution follows a specific spatial distribution pattern based on the plowing mode.

Soil microorganisms reveal the dynamics of soil structures. In this study, we found that microbial diversity differed in different plow layers. The bacterial analysis results of mulching with plastic film 0–20 a showed that the ACE and Shannon indices of soil samples in the 0–150 mm plow layer were significantly higher than those of soil samples in the 150–300 mm plow layer (P < 0.05), and that the abundance and diversity of bacterial communities declined with increasing soil depth, However, in the control group, bacterial communities became more abundant and diverse as the soil depth increased, which is consistent with the results obtained by Zhang et al. (2019b); who showed an upward trend with increasing water control depth. According to the results of fungal analysis, the abundance of fungal communities in the soil of the planting group decreased with increasing soil depth. According to this analysis, fertigation was performed in the cotton-planting area. With an increase in fertigation time, the nutrients finally converged via the interstices of the soil at a depth of 150–300 mm, where the soil is strongly fertile. Therefore, the microbial diversity of the soil in the control group, without film mulching, showed a similar trend. Overall, our results showed that the abundance and diversity of bacteria and fungi differed in typical areas at different tilling depths. To explore why the variation trend of film mulching planting was opposite to that of the control group, the influence of film mulching on the structure of microbial communities in the soil at different depths was further investigated.

To study the influences of film mulching on microbial communities in soils at different depths, Zhang et al. (2019a) studied the formation of a “microplasticizer” hot zone and concluded that the plastic is a “special microbial accumulator” in farmland soil that contains rich polyethylene-degrading groups, such as Actinomycetes, Bacteroides, and Proteus. In this study, Proteobacteria, Acidobacteria, Chloroflexi, Bacillus, and Actinobacteria were the dominant bacterial phyla in each treatment. Long-term film coating increased the relative abundance of Acidobacteria and reduced that of Proteobacteria and the relative abundance of Chloroflexi. The total number of OTUs, ACE index, Chao index, Simpson index, and Shannon index of the 0–150 mm cotton field mulched with film for 10–25a were higher than those of the control group. The residual film and microplastic form a physical barrier in the soil layer, which will eventually affect the downward penetration of water, fertilizer, and nutrients in the soil environment, thus affecting the root contact and absorption efficiency and affecting the microbial growth environment, which agrees with the conclusion reached by Wang (2016) in a study on the influences of residual mulching films on crop growth and microbial diversity: the soil samples mulched with films contained the most dominant bacterial flora, while those without film mulching contain the least dominant bacterial flora. In this study, the bacteria in the 0–150 mm plow layer in the control group were significantly less diverse than those in the 150–300 mm plow layer. However, as the number of planting years increased from 10 to 25a, there was no significant difference between the diversity of soil bacteria in the 0–150 mm and 150–300 mm plow layers of cotton fields with a planting life of 10 a; in the cotton fields with a planting life of 25a, the bacteria in the 0–150 mm plow layer were significantly more diverse than those in the 150–300 mm plow layer. With the increase in mulching years, from the 0–150 mm plow layer, the relative abundance of Proteobacteria declined significantly, whereas that of Actinobacteria and Chloroflexi increased significantly compared with the control group. In the 150–300 mm plow layer, the relative abundance of Proteobacteria was significantly higher than that of the control group, whereas that of Acidobacteria was significantly lower than that of the control group, which is consistent with the conclusion obtained by Zhao and Luo (2018); that is, the number of soil microorganisms decreased with increasing mulching years, with the number of Actinobacteria experiencing the most obvious change. Regarding the fungal communities in cotton fields, Ascomycota, Basidiomycota, and Mortierellomycota were the dominant phyla; film mulching remarkably reduced the relative abundance of Mortierellomycota, but significantly increased the relative abundance of Ascomycota. This is in line with the research findings obtained by Shen et al. (2016), that is, film mulching can increase the accumulation of nitrogen and enrich Ascomycota in mulching films. In this study, with increasing film-mulching years, microbial diversity showed a downward trend, with the number of microorganisms in the soil mulched with films being lower than that in the control group. This is relatively consistent with Gu et al.’s (2012) research findings on the diversity of microbial communities in the rhizosphere soil of cotton plants with different continuous cropping years in oasis farmlands in Xinjiang. The wasteland had the lowest number of microorganisms. The Shannon index of microbial communities gradually decreased when the continuous cropping period exceeded 15 years. This study showed that, with an increase in film mulching years to 25 years, the abundance of bacteria and fungi in the 0–150 mm and 150–300 mm cotton fields was significantly different, with bacteria being more diverse than fungi.

The residual mulching films in the soil of cotton fields gradually fragment, settle, and then scatter in layers in the 0–300 mm topsoil, which will affect the growth of the seeds by degrading the soil structure and destroying the soil environment. Over time, damaged films in the topsoil form microplastics (plastics with a diameter of less than 5 mm) and migrate to the soil, causing unknown impacts on crops and ecological security. Wang et al., 2020a, Wang et al., 2020b used 16S rRNA gene light sequencing to analyze the influences of microplastics on soil microorganisms, and the results showed that microplastics can improve the succession time of microbial communities and change soil microbial communities. Shi et al. (2014) found that residual mulching films can decrease soil porosity and permeability, reduce air circulation, weaken the distribution of microbial communities, worsen the survival conditions of the microbial population, and destroy the soil structure. This study showed that as the residual quantity of mulching films increased, the abundance of bacterial communities in the soil gradually declined. The residual mulching films had no significant effect on the microbial diversity of fungal communities, but they did affect the relative abundance of fungal communities in the soil of the cotton fields. This implies that the increased residual quantity of mulching films hinders the nutrients and fertilizers from migrating vertically, and the expansion of residual mulching films or microplastics enriches microorganisms, resulting in the phenomenon that microbial communities in different layers of soil differ with the prolonged mulching years and the increased residual quantity of mulching films.

## 5 Conclusion

To date, few studies have been conducted on the effects of residual films and microplastics on soil microbial diversity after long-term film mulching. In this study, an alar cotton field in a typical film-covered planting and reclamation area in Xinjiang was considered as an example. The analysis of the residual film showed that the residual film in a typical area had spatial distribution characteristics of 60–70% of the residual film in the 0–150 mm layer and 40–60% of the residual film in the 150–300 mm layer. Further research on soil microbial diversity using high-throughput sequencing technology showed that Proteobacteria, Acidobacteria, Campylobacter, Blastomonas, and Actinobacteria were the dominant bacteria in each treatment; Ascomycetes, Basidiomycetes, and Mortierella were the dominant fungi. According to the analysis of soil microorganisms at different depths under film-mulching planting conditions, the abundance and diversity of soil bacteria and fungi in the 0–150 mm soil layer were higher than those in the 150–300 mm soil layer, and the abundance and diversity of microbial communities decreased with increasing depth. Through the analysis of soil samples with different film-covering years, it was observed that when the film-covering years increased to 25 years, the residual amount of residual film and microplastic was enriched, and the microbial abundance of the bacterial community will decreased with the increase in residual film, but the impact on the fungal community was not significant. In short, the accumulation of residual film and microplastics has an impact on the diversity and community structure of farmland soil microorganisms, thus indirectly affecting the growth of soil aggregates and cotton. Therefore, while plastic films are convenient for agricultural production, attention should be paid to the impact of plastic films and microplastic residues on the farmland ecological environment.

## Declarations

### Author contribution statement

Jianfei Xing, Can Hu, and Xufeng Wang: Writing - Original Draft, Ideas, formulation or evolution of overarching research goals and aims, Preparation, creationand/or presentation of the published work, specifically visualization/data presentation;

Zhengxin Xu and Zaibin Wang: Conducting the research and investigation process, specifically performing the experiments, or data/evidence collection;

Long Wang, Xiaowei He, and Pengfei Zhao: Provision of study materials, reagents, materials, laboratory samples, instrumentation, computing resources, and other analysis tools;

Qi Liu: Management activities to annotate, scrub data, and maintain research data for initial use and later subsequent reuse.

Can Hu and Xufeng Wang: Conceptualization, Project Administration, Resources, Funding Acquisition, Supervision, and Writing - Review and Editing.

### Funding statement

Dr. Can Hu was supported by 10.13039/501100001809National Natural Science Foundation of China [32060288], the Bingtuan Science and Technology Program [2021CB010].

Xufeng Wang was supported by 10.13039/501100001809National Natural Science Foundation of China [32160300].

### Data availability statement

Data associated with this study has been deposited at the Sequence Read Archive (SRA) of the National Center for Biotechnology Information (NCBI) under the accession number SRP392419 under project PRJNA869864.

### Declaration of interest's statement

The authors declare no conflict of interest.

### Additional information

Supplementary content related to this article has been published online at https://doi.org/10.1016/j.heliyon.2022.e12180.

## Appendix A Supplementary data

Supplementary Figure.docx Supplementary Figure.docx

Supplementary Table.docx Supplementary Table.docx
