# Supplementary material for: Effects of residual mulching films with different mulching years on the diversity of soil microbial communities in typical regions
Source: Heliyon. 2022 Dec 12;8(12):e12180. doi: 10.1016/j.heliyon.2022.e12180 (PMC9791357; doi:10.1016/j.heliyon.2022.e12180)
Supplement: Supplementary Figure.docx [file mmc1.docx]

| 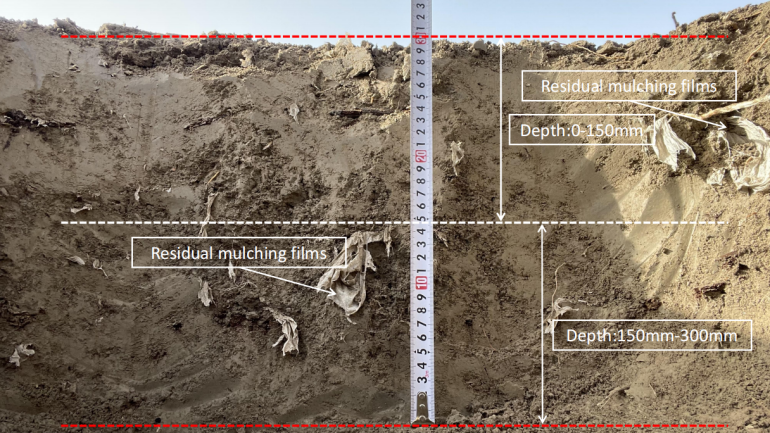 |
| --- |
| **Fig. S1.** Sectional analysis |

| 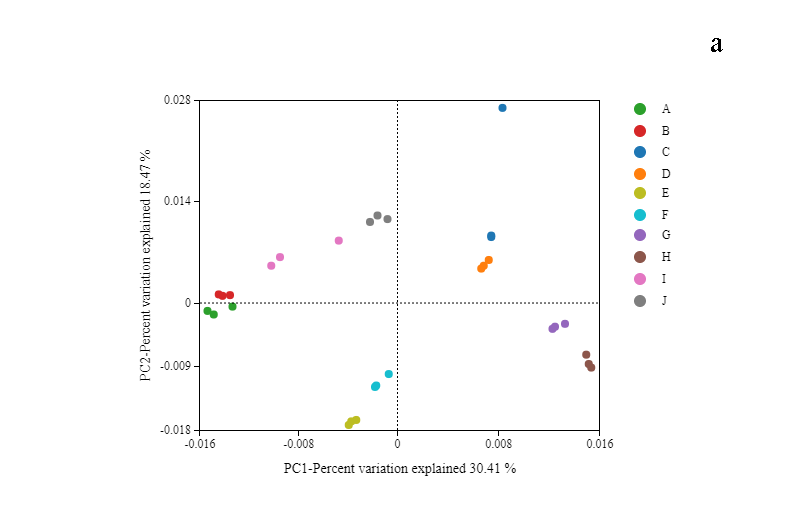 | 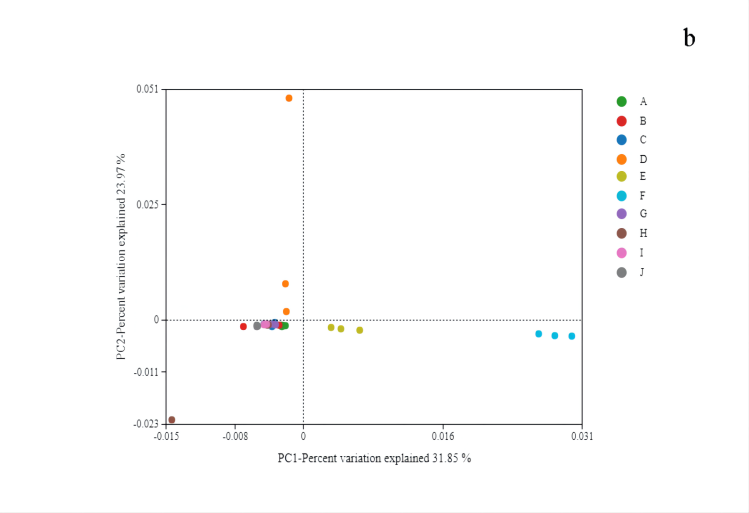 |
| --- | --- |
| **Fig. S2.** Principal coordinate analysis results of microbial community structures in different soil layers.Results of principal coordinate analysis of the structure of 0-150mm and 150-300mm biome in soil bacteria and fungi. (a) Principal coordinate analysis of soil bacteria. (b) Principal coordinate analysis of soil fungal. | |

| 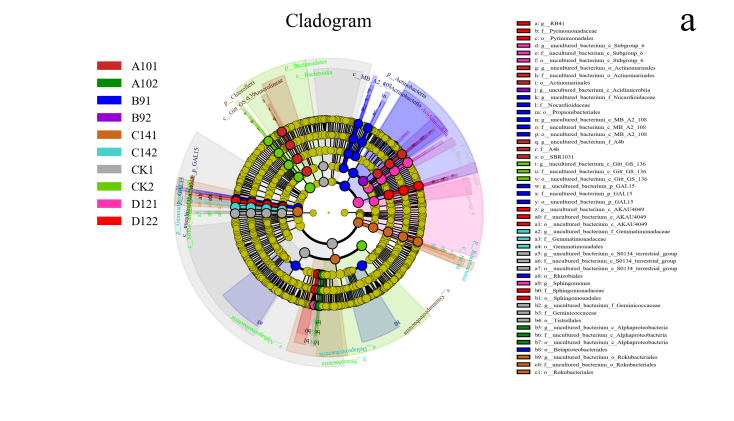 | 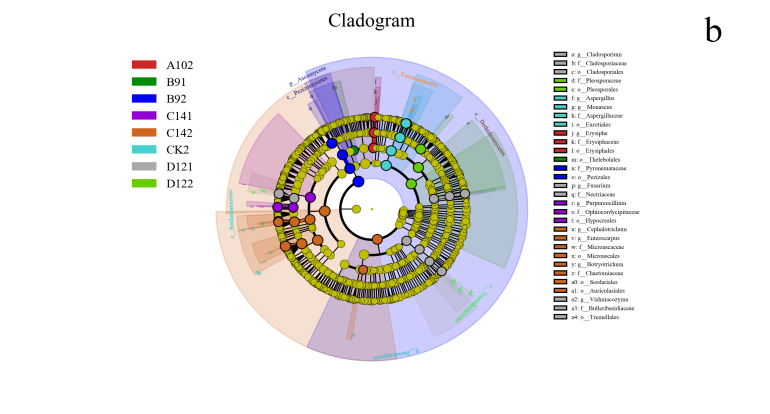 |
| --- | --- |
| **Fig. S3.** LefSe analysis of microbial communities in soil. (a) LefSe analysis of soil bacterial communities; (b) LefSe analysis of soil fungal communities. | |

| **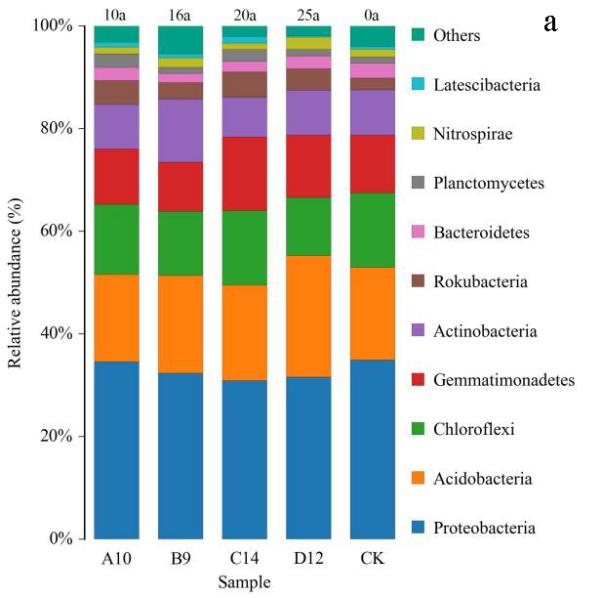** | **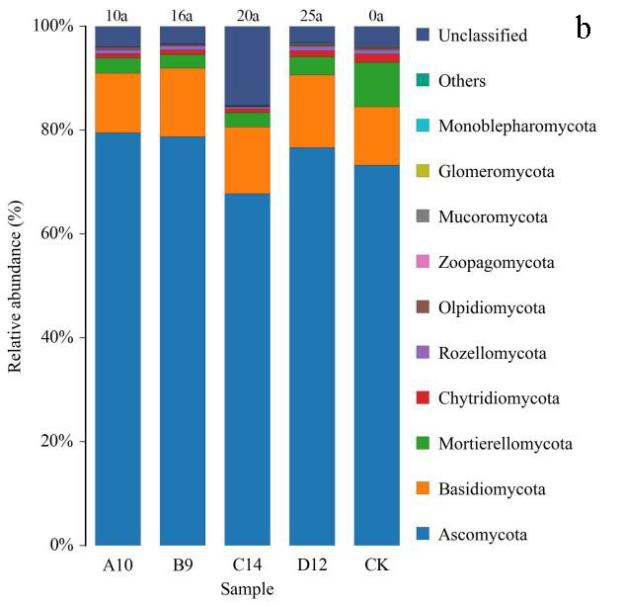** |
| --- | --- |
| **Fig. S4.** Composition and Relative Abundance of Soil Microorganisms at phylum Level with Different Residual Film Amount. (a) Soil bacterial. (b) Soil fungal. | |
