# Supplementary material for: Effects of residual mulching films with different mulching years on the diversity of soil microbial communities in typical regions
Source: Heliyon. 2022 Dec 12;8(12):e12180. doi: 10.1016/j.heliyon.2022.e12180 (PMC9791357; doi:10.1016/j.heliyon.2022.e12180)
Supplement: Supplementary Table.docx [file mmc2.docx]

**Table S1** Distribution of the average amount of residual mulching films at each sampling site

| Depth | The No. 10 farm（kg/hm^2^） | The No. 9 farm（kg/hm^2^） | The No. 14 farm（kg/hm^2^） | The No. 15 farm（kg/hm^2^） |
| --- | --- | --- | --- | --- |
| 0-150mm | 26.6 | 43.7 | 62.4 | 114.3 |
| 150-300mm | 18.1 | 28.3 | 33.5 | 47.9 |
| Total | 44.7 | 72 | 95.9 | 162.2 |
| Planting years（a） | 10 | 16 | 20 | 25 |

Residual film residues of 0-150mm and 150-300mm at each sampling point (kg/hm^2^).
